# Austrian male patients’ gender role conflict is associated with their wish for interpersonal violence to be addressed during patient-physician conversations: a questionnaire study

**DOI:** 10.1186/s12889-020-09436-4

**Published:** 2020-08-27

**Authors:** Nikola Komlenac, Heidi Siller, Margarethe Hochleitner

**Affiliations:** 1 Gender Medicine Unit, Medical University of Innsbruck, Fritz-Pregl-Strasse 3, 6020 Innsbruck, Austria; 2 Gender Medicine Unit, Medical University of Innsbruck, Innrain 66, 6020 Innsbruck, Austria

**Keywords:** Men, Masculinity, Interpersonal violence, Healthcare, Gender role conflict, Patient-physician conversation, Austria

## Abstract

**Background:**

Experiencing interpersonal violence and disclosing this experience to physicians can be associated with fear, shame, denial or emotional turmoil. Expressions of such feelings additionally conflict with masculine gender role ideologies and may be experienced as masculine gender role conflict. Masculine gender role conflict is often associated with men’s unwillingness to seek professional help. The current study analyzed the association between masculine gender role conflict and men’s wish for patient-physician conversations to include questions about interpersonal violence they might have experienced.

**Methods:**

In structured closed-ended interviews conducted at an Austrian hospital 129 male patients (*M*_age_ = 59.4, *SD* = 14.7) were asked what forms of interpersonal violence they had experienced. Additionally, a study-specific questionnaire was used to find out whether male patients wanted future physician-patient conversations to include questions about interpersonal violence they might have experienced. Men’s gender role conflict was assessed with the Gender Role Conflict Scale-Short Form.

**Results:**

Half of the male patients (50%) reported having experienced at least one form of interpersonal violence. Nearly half of the men (48%) wanted their physician to ask them in future about any violence they might have experienced. One pattern of gender role conflict was positively associated with men’s wish to be asked in patient-physician conversations about potential interpersonal violence. Namely, men who reported conflicts between work and family relations were more likely to state that they wanted such conversations (*OR* = 1.6, 95%CI = 1.1–2.4) than were men who did not often experience this pattern of gender role conflict.

**Conclusions:**

Experiences of interpersonal violence should be an important part of physician-patient conversations with male patients. Overall, male patients would welcome their physician initiating a potential conversation about violence. Using an approach that takes consideration of masculine gender role ideologies may further increase some men’s willingness to approach the topic of interpersonal violence. Men who adhere to the norm of being preoccupied with work may be more willing to talk about this subject if healthcare situations are framed in a way that men perceive the possibility to uphold masculine gender role ideologies of self-sufficiency or of being in control.

## Backgroud

Interpersonal violence is a person’s intentional use of physical force or power directed towards a family member, intimate partner, an unrelated individual or against an acquaintance or friend [1]. Interpersonal violence can take many forms. Threats or acts of physical violence, sexual violence, and psychological violence are forms of interpersonal violence [1]. Next to variable physical injuries and physical traumata [2], other medical conditions, diseases and mental health problems that can even lead to disability or death can be consequences of interpersonal violence [3].

Studies conducted in men report that a considerable proportion of men are victims of interpersonal violence. For instance, a population based study in Germany reports that 6.2% of men had experienced interpersonal physical violence and 17.3% of them were affected by interpersonal psychological violence in the previous 12 months [4]. Similarly, in Sweden in a study among young people, 9.7% of men reported having experienced physical violence during the previous 12 months [5]. In a Finish study, more than half (57.1%) of the participating young adult men reported having experienced some kind of physical violence or having been threatened with bodily harm or property damage in their lifetime [6]. Such high prevalence rates for experienced interpersonal violence are also reported in Austria [7]. A population-based study in Austria revealed that 61.4% of men had experience interpersonal physical violence, 78.4% of them had experienced interpersonal psychological violence, and 8.8% of Austrian men had experienced sexual violence during their adulthood. It is of note that of the experiences of interpersonal physical violence, most happened in public spaces [5, 7] or were committed by an unrelated person [4].

Even though many of those men who experienced interpersonal violence suffer negative physical or mental health consequences [8–10], men are in general reluctant to seek formal or medical help after experiencing violence [11–13]. The Model of Pathways to Treatment [14, 15] explains people’s help-seeking behavior. According to this model, people need to perceive a reason to seek help from a healthcare provider (appraisal interval). After perceiving a need for help, people may delay or avoid medical help-seeking because of perceived barriers during the so-called help-seeking interval [14, 15]. Barriers that influence help-seeking during the appraisal or help-seeking interval include normalizing or not taking the symptoms seriously [16], not knowing about treatment or service options, high costs of medical services, or feelings of embarrassment, anxiety, or fear [17, 18]. Some unique barriers to help-seeking after the experience of interpersonal violence are the fear of repercussions or the fear of being stigmatized for being a victim of violence [19].

Help-seeking after experiencing interpersonal violence may additionally be avoided by men because of masculine gender role conflict. Masculine gender role conflict is a form of distress that men who endorse masculine gender role ideologies may experience when behaving in a manner that does not uphold masculine gender role ideologies [20]. Masculine gender role ideologies are socially defined descriptions of behaviors, thoughts or attitudes that are associated with male sex and are based on a culture’s gender-role stereotypes and gender role norms [21–23]. Traditional masculine gender role ideologies originated in the late 1960s. Those ideologies depict men as being aggressive, physically strong, and needing to defend one’s interests with physical force. At the same time men are depicted as being unlikely to be psychologically affected by experiences of interpersonal violence [24]. Additionally, being dependent on another person or needing help violates those masculine ideologies. Therefore, the experience of being a victim of interpersonal violence or needing to seek help after experiencing interpersonal violence are in conflict with masculine gender role ideologies [24] and help-seeking may be experienced as a gender role conflict.

Accordingly, previous studies found that the experience of gender role conflict is associated with reduced willingness for psychological and medical help-seeking [17]. Mostly, it has been found that men who are distressed when expressing intimate emotions (which would violate the traditional masculine ideology of men being stoic) are more likely to hold negative attitudes towards psychological help-seeking [25] than are men who rarely experience such a gender role conflict. Also other patterns of gender role conflict, such as distress when showing positive affection towards other men or a focus on success, power, and competition have been found to be positively associated with negative attitudes towards psychological help-seeking [26]. With regard to medical help-seeking, distress experienced when expressing intimate emotions has been linked to men’s unwillingness to seek medical help for psychological symptoms [27] or sexual problems [28].

To date, no study has investigated the association between masculine gender role conflict and men’s willingness to talk or their wish to be asked about potential experiences of violence during patient-physician conversations. Qualitative studies support the hypothesis that gender role conflict may be negatively associated with men’s wish for patient-physician conversations to include questions about interpersonal violence. In qualitative studies men who were attempting to adhere to masculine gender role ideologies reported that it was not likely that they would divulge their experience of interpersonal violence to a healthcare professional [13]. Especially men’s tendency to restrict emotional expressivity and their reluctance to talk about emotional and intimate issues seemed to influence men’s willingness to talk or their wish to be asked about violence they might have experienced [29].

Because of the many barriers faced during the appraisal or help-seeking interval, men may delay or avoid help-seeking altogether for interpersonal violence [14, 15]. This avoidance or delay of help-seeking can have negative consequences, because interpersonal violence is a relevant health problem that needs to be addressed (e.g., [30]). Physicians may help some men shorten or omit those intervals by routinely asking all patients about potential experiences of interpersonal violence (irrespective of patients’ reasons for presentation). This way at least male victims of interpersonal violence who are already seeing a physician (for another reason) would get the opportunity to disclose potential experiences of interpersonal violence and would be more likely to receive help, if needed. However, physicians rarely ask their patients for interpersonal violence experiences [31, 32]. Reasons for the small number of patient-physician conversations that include questions about potential experiences of interpersonal violence are, in addition to a lack of time, a lack of training or a lack of knowledge about referral routes. Physicians also often fear their patients would retaliate or be offended by such an approach [32–34]. Many physicians additionally think that their patients would bring the topic up themselves if they felt the need to talk about such issues [31, 32].

Only in recent years were some studies conducted on men’s views about being asked by a healthcare practitioner about violence they might have experienced. The focus of these studies was on domestic violence [29, 35]. One study in the UK found that only 1.6% of the survey’s male participants had been asked by a healthcare professional about domestic violence they might have experienced and only 8% of the men thought a healthcare professional should not ask patients about domestic violence they might have experienced [29]. The literature lacks men’s views about discussing interpersonal violence and not specifically domestic or intimate partner violence. This is relevant because men are more often affected by interpersonal violence that is not perpetrated by a family member or intimate partner [4]. Consequently, the focus on domestic or intimate partner violence concerning men’s help-seeking does not consider the broad spectrum of interpersonal violence or men’s help-seeking intentions.

## Aim of the study

To date, studies on men’s views about being asked by physicians about interpersonal violence they might have experienced are missing. Previous studies on this topic have focused on domestic violence [29, 35]. Therefore, the current study examines interpersonal violence that includes domestic violence, but extends beyond domestic violence and includes additional facets of violence [1]. This study assessed the prevalence of experienced interpersonal violence among male in-patients at a medical university hospital in Austria and those male patients’ views on whether in a healthcare setting they want their physicians to ask them about potential experiences of interpersonal violence. Additionally, we included a measure of men’s gender role conflict in the current study, because experiences of gender role conflict are associated with men’s views on help-seeking [26]. Additionally, qualitative studies have shown that the degree to which a man has internalized masculine gender role ideologies can influence his opinion on help-seeking for experienced violence [36]. Our research questions (RQs) of the current study follow:

- RQ1: How many male in-patients at a medical university hospital in Austria report experiences of interpersonal violence in the past?
- RQ2: Do male patients want their physicians to routinely include questions about potential experiences of interpersonal violence during patient-physician conversations?
- RQ3: Do men who experience gender role conflict have more negative views on discussing interpersonal violence during patient-physician conversations than do men who seldom experience gender role conflict?

## Methods

### Procedure

Between September and December 2016 successive male in-patients at a medical university hospital in Austria at the Department of Internal Medicine who were older than 18 years and who were able to speak and understand German were invited to participate in the study. The participants in the present study had previously also participated in another already published study. Thus, the detailed procedure can be found elsewhere [28]. All patients approached were verbally informed that the study was about sexual health [28] and past experiences of interpersonal violence and whether such rarely discussed topics should be subject of routine patient-physician conversations. All participants were offered a list of relevant services for help after having experienced violence or sexual health problems. The current study is the first to utilize these participants’ responses to questions about the topic of violence.

The Medical University’s Ethics Committee (ID: AN2016–0093 362/4.5) approved the study. The study was conducted in accordance with the Declaration of Helsinki [37] and the APA standards [38]. All participants gave written informed consent. Participation was voluntary and patients could withdraw their consent or refuse participation at any time without any negative consequences.

### Measures

This study used two methods of data collection [28]. The first part employed a structured face-to-face interview with closed-ended answer categories. Participants were asked about demographic variables and experienced violence. A structured face-to-face interview approach was chosen to enable patients to experience how a conversation about experienced violence feels before stating whether they want their physician to ask them about such a topic. The same male interviewer (NK) conducted all interviews [39]. During the interview the interviewer wrote participants’ answers down or noted answers by checking predefined answer categories. The second part of the study involved a paper-pencil questionnaire. This paper-pencil questionnaire included questions about previous discussions with a physician concerning experienced violence, the wish to be asked in future by a physician about possibly experienced violence and a validated questionnaire about gender role conflict.

#### Demographic variables

Participants were asked for their age, nationality (*Austrian* vs. *German* vs. *Turkish* vs. *Italian* vs. *other*), highest level of education (*secondary school* vs. *vocational school* vs. *university entrance-level qualification* vs. *university degree*), sexual orientation (*heterosexual* vs. *bisexual* vs. *gay-identified*) and relationship status (*single* vs. *with partner*). The variables nationality and sexual orientation were not considered beyond descriptive analyses because of the majority of participants being Austrian or heterosexual-identified. For the variable highest level of education a dummy variable was formed by merging the categories *secondary school* and *vocational school* into a new category.

#### Experienced violence

A structured interview was developed for the current study, because validated structured interviews assessing interpersonal violence (e.g., [40]) do not assess distinct forms of interpersonal violence [1] separately. The interview was developed based on a literature search [6, 29, 35] and assessed various forms of interpersonal violence as categorized by the World Health Organization [1]. Patients were asked, “Did you experience the following form of violence in the past?” Each of the following five forms of violence were assessed separately: physical violence, sexual abuse, verbal abuse, repeated humiliation and serious threats. A specific report of violence was followed by a question to specify what experience was referred to and whether participants experienced the reported violence as being threatening or serious. A dichotomous variable was formed for each of the five forms of experienced violence (*no* vs. *yes*). All participants who were coded as having had an experience of violence indicated that they referred to severe acts of abuse that they perceived as frightening or as serious threats [35].

#### Discussions with a physician about experienced interpersonal violence

Patients were asked, “In the past, how often have you been asked by a physician about potential experiences of interpersonal violence?” Responses could be given on a four-point Likert scale (*never* vs. *rarely* vs. *sometimes* vs. *often*). Because only four participants responded that they had been asked such questions by a physician in the past, a dichotomous dummy variable was used to differentiate between men who had *never* been asked by a physician about experienced interpersonal violence and those who had been at least *rarely* asked by a physician about such issues. Moreover, patients were asked whether they would like their physician to ask them about interpersonal violence they had possibly experienced. They could respond *no* vs. *yes* to the question, “Would you like your physician to ask you about potential experiences of violence?” A similar approach was used by Bacchus, Buller [35].

Furthermore, patients were asked how they wanted the topic of violence to be addressed by a physician in more detail. They were asked, “In future, how would you like the topic of interpersonal violence to be handled during patient-physician interactions?” Patients could choose one of four responses that best indicated how they wanted the topic of violence to be addressed by a physician in future. This question was modified from previous studies on this topic [35, 41]. The first response option was, 0 = “For the future, I would like physicians not to talk about violence.” The second option was developed for the current study based on assumptions that physicians often hold, according to which patients would bring the topic up themselves if they felt the need to talk about experiences of violence [31, 32]. The second options was, 1 = “For the future, I would like physicians to wait until I bring up the topic of violence.” The third response option was developed for the current study, because one known reason for delaying help-seeking during the help-seeking interval is a person’s uncertainty about whether they have a “legitimate” concern to present to a physician [14, 15]. The third response option therefore was, 3 = “For the future, I would like physicians to say that it would be legit to talk about violence with them, but then wait until I want to talk more about this topic.” The fourth response option was adapted from previous studies [35, 41] and was 4 = “For the future, I would like physicians to directly ask me and talk about the topic of violence.”

#### Gender role conflict

Gender role conflict was assessed with the Gender Role Conflict Scale - Short Form (GRCS-SF) [20]. Permission to use the GRCS-SF in the current study was obtained from the original author James M. O’Neil on 12 April 2016. This questionnaire consists of four scales, each assessing one of four patterns of masculine gender role conflict [42]. Gender role conflict is caused when a person experiences distress, stress or discomfort in situations in which they behave in a way that conflicts with prescribed masculine gender role ideologies [20, 42]. One pattern of gender role conflict is success, power, and competition. The prescribed masculine norm behind this pattern is the norm of men needing to be constantly obsessed with being better and more successful than other people. People who are distressed when expressing intimate emotions show a different pattern, called restrictive emotionality. Men who show the pattern of restrictive affectionate behavior between men refrain from showing positive affection towards other men. For men who show the pattern conflict between work and family relations it is difficult to find enough time for their family or leisure activities because of being preoccupied with their work [20, 42]. Each scale consists of four items. Men were asked to indicate the degree of experienced conflict on a six-point Likert scale ranging from 0 (*strongly disagree*) to 5 (*strongly agree*). Scale scores were the mean value of the items belonging to a certain scale. High scores indicated frequent gender role conflict. In the current study all scales had acceptable internal consistencies (Table 1). Previously reported Cronbach’s αs ranged from .77 to .80 [20]. The four-factor structure of the German-language version of the GRCS-SF was previously confirmed [43].

**Table 1:** Correlations between the Gender Role Conflict Scale - Short Form Scales and Male Patients’ Wish to be Asked by a Physician about Potential Experiences of Interpersonal Violence (*N* = 129)

	1.	2.	3.	4.	5.
1. CBWFR	–	.34**	.25**	.36**	.21*
2. RE		–	.39**	.40**	.02
3. RABBM			–	.34**	−.16
4. SPC				–	−.10
5. Wish to be asked					–
*M* (*SD*)	2.3 (1.2)	1.6 (1.1)	2.0 (1.4)	1.8 (1.1)	
Cronbach’s α	.67	.74	.77	.73	

*Note*. *CBWFR* Conflict Between Work and Family Relations, *RE* Restrictive Emotionality, *RABBM* Restrictive Affectionate Behavior Between Men, *SPC* Success/Power/Competition. *p < .050, **p < .010

### Statistical analysis

This exploratory study assessing violence experienced by men and their wish for patient-physician conversations about the potential experience of violence presents many results with descriptive statistics. Correlations and logistic regression models were calculated to estimate the association between gender role conflict and patients’ wish for patient-physician conversations to include questions about experienced violence.

Hierarchical logistic regression models were calculated [44]. Demographic information (age, education, relationship status) and experienced violence were first entered as a group. In the second step all four scales of the GRCS-SF were entered as a group. Effect sizes (Nagelkerke *R*^2^) are reported for each overall regression model. Odds ratios (*OR*) with a 95% confidence interval (*CI* [range]) are reported.

The level of significance for all analyses was α = .05. All statistical analyses were performed with the Statistical Package for the Social Sciences (SPSS) for Windows, version 25.0.

## Results

### Participants

In total, 253 male in-patients were invited to participate in the study. Of these men 133 participated (response rate = 52.6%), whereby 129 patients provided full responses for analysis. On average, participants were 59.4 (*SD* = 14.7; range: 23–84) years old. The majority of participants (87.6%) had Austrian nationality. The remaining men came from either Germany (6.2%), Italy (3.1%) or other nations (3.1%). More than half (57.4%) of the sample stated that their highest level of education was secondary school or that they had finished vocational school. In the study there were nearly as many participants (22.5%) with a university entrance-level qualification as there were participants with a university degree (20.2%). Most men (76.7%) were in a relationship, whereas the other men (23.3%) were not in a relationship at the time of the study. The current sample consisted mostly of heterosexual-identified men (90.7%). The sample included equal percentages of bisexual men (1.6%) and gay-identified men (1.6%). The remainder of the sample did not respond to the question about sexual orientation (6.2%).

### Prevalence of experienced violence

Half of the sample (50.4%) reported having had experienced at least one form of serious interpersonal violence in the past. Of the men who had experienced at least one form of violence, 46.2% of them had experienced more than one form of violence. The most common form of violence experienced was physical violence (29.5%). A similar number of men reported being verbally abused (25.6%). Of the participants, 18.6% were seriously threatened in the past. Repeated humiliations were reported by 12.4% of the patients. Two men (1.6%) disclosed past sexual abuse.

### Discussions with a physician about experienced violence

Only four (3.1%) participants had been asked by their physician about experienced interpersonal violence. All these men had experienced at least one form of violence in the past.

Nearly half (48.1%) of all participants stated that they would like their physician to ask them about interpersonal violence they may have experienced. In this regard, men who had experienced violence (51.4%) did not differ from men who did not report having experienced serious violence in the past (43.9%; χ^2^(1) = 0.72, *p* = .395).

Only eight men (6.3%) stated that in future they would like to never talk with a physician about experienced violence. Close to a fifth (22.2%) of the participants preferred to bring the topic up themselves. Most participants wanted their physician to initiate a potential conversation about violence. A quarter (25.4%) of all patients wanted their physician to introduce the topic of violence, legitimate such conversations and ensure that they would be supportive if the patient decided to initiate such a conversation. The remaining participants (46.0%) would like their physician to directly ask questions about violence they may have experienced.

### Gender role conflict

On average male patients reported having seldom experienced gender role conflict (Table 1). A significant correlation was seen between the conflict between work and family relations and a patient’s wish to be asked by a physician about interpersonal violence he may have experienced (Table 1). This relationship remained in the logistic regression model while controlling for age, education, relationship status, experienced violence, and other patterns of gender role conflict (Table 2). Participants who often experienced conflict between work and family relations were more likely to state that they wanted questions about experienced violence to be part of the patient-physician conversation than were men who rarely experienced this gender role conflict. Even though the focus on success, power, and competition nearly proved to be uniquely related with a patient’s wish to be asked by a physician about interpersonal violence he may have experienced (*p* = .086), no other pattern of gender role conflict had a significant association with this variable (Table 1, Table 2).

**Table 2:** Hierarchical Logistic Regression Model Predicting whether Male Patients Wish to be Asked by a Physician about Potential Experiences of Interpersonal Violence (*N* = 129)

Variables	*B*	*SE B*	*OR*	95% CI for *OR*		*R*^2^	Δ*R*^2^
				*LL*	*UL*		
Step 1						0.09	0.09
Constant	1.41	1.23					
Age	−0.04**	0.01	0.97	0.94	0.99		
Education	−0.04	0.23	0.96	0.60	1.51		
Single/in relationship	0.40	0.44	1.49	0.62	3.55		
Past experiences of violence^a^	−0.08	0.38	0.92	0.44	1.93		
Step 2						0.19	0.10*
Constant	0.99	1.37					
Age	−0.03^†^	0.01	0.97	0.95	1.00		
Education	−0.09	0.26	0.92	0.55	1.52		
Single/in relationship	0.26	0.47	1.30	0.52	3.27		
Past experiences of violence^a^	−0.08	0.40	0.92	0.43	2.00		
CBWFR	0.49*	0.19	1.63	1.12	2.39		
SPC	−0.35^†^	0.21	0.71	0.47	1.07		
RABBM	−0.26	0.16	0.77	0.56	1.06		
RE	0.14	0.21	1.15	0.76	1.73		

*Note*. *CBWFR* Conflict Between Work and Family Relations, *SPC* Success/Power/Competition, *RABBM* Restrictive Affectionate Behavior Between Men, *RE* Restrictive Emotionality, *CI* Confidence interval, *LL* Lower limit, *UL* upper limit. ^a^0 = no past experiences of interpersonal violence, 1 = at least one form of serious violence in the past.^†^*p* < .100. *p < .050. **p < .010

## Discussion

The current study revealed that half of the male patients who participated in the study reported having had experienced at least one form of serious interpersonal violence in the past (RQ1). Half of male patients would like their physician to routinely ask them about interpersonal violence they might have experienced (RQ2). Masculine gender role conflict was associated with patients’ views on whether they wanted their physicians to actively ask them about interpersonal violence during patient-physician conversations (RQ3).

### Patients’ wish that violence be addressed by a physician

The current study adds further evidence that the experience of violence is prevalent in men [4–7]. Half of the current participants reported having experienced at least one form of interpersonal violence in the past. Physical and psychological violence were the most frequent forms of violence reported by men, comparable to results from other studies (e.g., [7]). Because the experience of interpersonal violence often leads to negative physical or mental health consequences [2, 3], and because of the high prevalence rates of affected men, interpersonal violence should be an important issue in medical healthcare (e.g., [30]).

Irrespective of this need for patient-physician conversations about violence, physicians are often reluctant to address the subject of violence their patient might have experienced [31, 32]. For example, only four participants of the current study had talked with their physician about potential or actually experienced interpersonal violence in the past. However, the current study and previous research [29, 35] further revealed that many male patients would like their physicians to initiate the topic of interpersonal violence during patient-physician conversations. In our study nearly half of the patients wanted their physicians to directly and routinely ask them about interpersonal violence they had possibly experienced. Many patients wanted their physician to introduce the topic of violence, and thereby to legitimate such conversations.

During medical education or during continued training physicians should learn the importance of including questions about potential experiences of violence during patient-physician conversations and different ways to approach this topic with their patients. Furthermore, physicians should learn how to respond with empathy and how to document potential physical evidence of interpersonal violence. Physicians should know and be able to offer referrals to specialist services if needed [45, 46]. The fact that many patients welcome such conversations may additionally motivate many physicians to start implementing such conversations in everyday clinical practice.

### Masculinity and conversations about interpersonal violence

Many studies have found associations between men’s experience of gender role conflict and their willingness to seek help [17]. Especially the patterns of restricted emotionality, restrictions concerning showing positive affection towards other men, and the focus on success, power, and competition have been found to be negatively associated with men’s wish to seek psychological or medical help [25–28]. Qualitative studies reported that some men’s adherence to masculine gender role ideologies negatively influenced their willingness to talk about interpersonal violence or their wish to be asked by a physician about this topic [13, 29]. In contrast, no negative associations between men’s gender conflict and men’s wish to be asked by physicians about potential interpersonal violence were found in the current study. The current findings are supported by other studies that also did not find any negative associations between men’s help-seeking intentions and their experience of masculine gender role conflict [47–49]. A recent study suggests that the adherence to masculine gender role ideologies rather than the experience of distress when behaving in violation of those ideologies is associated with men’s help-seeking intentions [50]. Future studies are needed to clarify whether conformity to gender role ideologies rather than experiences of gender role conflict are negatively related to men’s wishes for patient-physician conversations to include questions about interpersonal violence.

Such future studies should additionally include a sample size larger than that of the current study, because the finding that no negative associations exist between men’s gender conflict and their wish to be asked by physicians about potential interpersonal violence may also have resulted from the current study’s relatively small sample size. Given the sample size and the number of variables, there were concerns that the study could be underpowered. One such association that could have been missed if the study was underpowered is be a negative association between men’s focus on success, power, and competition and their wish for physicians to ask them about potential experiences of violence. This association almost proved to be significant in the current study. Previous qualitative studies reported that men’s concerns about not appearing “weak” are a factor that influences men’s help-seeking intentions after experiences of interpersonal violence [51, 52]. Therefore, it may be important for physicians to carefully avoid communicating to their patients opinions that would imply that male victims of interpersonal violence are weak in order to not discourage some male victims of interpersonal violence from asking for help [13, 53].

One pattern of gender role conflict was positively associated with men’s wish to be asked in patient-physician conversations about potential interpersonal violence. As shown in this study, men who reported conflicts between work and family relations were more likely to state that they wanted such conversations than were men who did not often experience this pattern of gender role conflict. The underlying theme of this pattern of gender role conflict is the wish to be self-sufficient, in control, and being preoccupied with work [54]. Also in other studies these masculine themes have proven to be associated with positive aspects of health [48, 49, 55]. The current study therefore adds further evidence to show that some masculine gender role ideologies can be beneficial for some health issues.

This means that for some men it may help when healthcare situations are framed in a way that men perceive the possibility to uphold masculine gender role ideologies of self-sufficiency or of being in control [56]. Such a framing could emphasize men’s responsibility and control to change unfavorable health outcomes by seeking formal help [57, 58]. Health promotion and a thorough patient-physician conversation that considers all aspects of men’s health – including the possibility of having experienced interpersonal violence – can be approaches that are supported by some men who want to adhere to the ideology of being preoccupied with work [59].

However, there is no “one size fits all” approach that can be recommended when working with men and trying to increase their healthcare utilization [60, 61]. Only few men in the current study often experienced gender role conflict and they differ in their patterns of experienced gender role conflict. Physicians may discuss their patients’ decision to seek help and explore their patients’ experiences of gender role conflicts during such discussions [47]. Additionally, physicians can formally assess male patients’ gender role conflict with the Gender Role Conflict Scale - Short Form [20]. Using an approach that takes consideration of masculine gender role ideologies may further increase men’s already high willingness to approach the topic of interpersonal violence during patient-physician conversations.

### Limitations

Next to the already discussed small sample size, the current study has other limitations worth noting. First, the study is based on self-reported data about events that happened in the past. This approach can entail many problems. For one, the participants may not remember all incidences of experienced violence or past patient-physician conversations about this topic. Additionally, patients can choose what to report and what not to report. Information can willingly be withheld or divulged by a participant when he considers it to be socially desirable [62].

One more concern regarding social desirability and the withholding of information could be the face-to-face interview method. For example, men could have found it difficult to disclose past experiences of sexual abuse during the face-to-face interview. Felt shame [13], feelings of vulnerability [63], or the difficulty of acknowledging a sexual victimization experience [64] during the interview could have hindered men in the current study from reporting past experiences of sexual abuse. This may explain the smaller number of men who reported past experiences of sexual abuse in the current study as compared to prevalence rates found in previous studies (e.g., [7]). It has been reported that higher prevalence rates of past experiences of violence, especially of sexual abuse, are estimated when online questionnaires rather than face-to-face interviews are used to assess such experiences [7]. However, a face-to-face interview was chosen in the current study to enable patients to experience how a conversation about experienced violence feels before stating whether they want their physician to ask them about such a topic. Face-to-face interviews also have advantages. For instance, such interviews are known to have lower non-response rates than are self-administered study methods [65].

Secondly, the interview’s structured format did not allow patients to report any additional kind of experienced interpersonal violence. The list of interpersonal violence acts may not be exhaustive. Thus, some cases or forms of interpersonal violence may have been missed in the current study. However, the current questionnaire assessing interpersonal violence experiences was developed based on the available literature [6, 29, 35] and our aim was to broaden the questionnaire’s scope by not focusing solely on domestic or intimate partner violence. Nevertheless, the current questionnaire did not assess the affected men’s relationship with the perpetrator or whether men themselves were using violence when they experienced interpersonal violence. A next step toward increasing our understanding of men’s experiences of interpersonal violence and their help-seeking intentions would be to analyze whether men’s experiences and their help-seeking intentions differ depending on their relationship with the perpetrator or on whether they themselves were using violence when experiencing interpersonal violence.

The current sample consisted mostly of heterosexual-identified men. Therefore, the variable sexual orientation could not be considered in the logistic regression models and potential differences depending on men’s sexual orientation could not be analyzed. This is a considerable limitation because men’s experiences may differ depending on their sexual orientation (e.g., [66, 67]) and their view of patient-physician conversations may also differ accordingly. Gay-identified men or other not heterosexual-identified men may especially face issues of homophobia and heteronormativity when seeking medical help for interpersonal violence [68].

## Conclusions

Experiences of violence are an important health issue that should be addressed in healthcare [3, 30, 69]. In the current study, half of the male patients had experienced at least one form of interpersonal violence. Most patients would welcome the topic of interpersonal violence brought up during patient-physician conversations. Only few male patients wished to never again talk about interpersonal violence in the clinical setting. The current study supports the claim that some masculine gender role ideologies may be beneficial for men’s willingness to obtain medical help or support with regard to experienced violence. Namely, men who internalized the masculine gender role ideology of putting work above all other life priorities and who as a result experienced conflict between work and family relations were more likely to advocate patient-physician conversations about potentially experienced interpersonal violence than were men who did not experience this pattern of gender role conflict. Physicians may try to use men’s adherence to masculine gender role ideologies to best advantage by reframing the healthcare situations for those men who adhere to the norm of being preoccupied with work so that the situations give them a sense of control and of being self-sufficient. Thus, these men may utilize healthcare offers that also include patient-physician conversations about interpersonal violence, while at the same time they are able to maintain their sense of masculinity.

## Data Availability

The datasets used and/or analyzed during the current study are available from the corresponding author on reasonable request.

## Abbreviations

GRCS-SF: Gender Role Conflict Scale-Short Form
UK: United Kingdom
RQ: Research question
APA: American Psychological Association
OR: Odds ratio
CI: 95% confidence interval
